# Predicting sepsis mortality into an era of pandrug-resistant *E. coli* through modeling

**DOI:** 10.1038/s43856-024-00693-7

**Published:** 2024-12-26

**Authors:** Benjamin J. Koch, Daniel E. Park, Bruce A. Hungate, Cindy M. Liu, James R. Johnson, Lance B. Price

**Affiliations:** 1 Center for Ecosystem Science and Society, Northern Arizona University, Flagstaff, AZ USA; 2 Department of Biological Sciences, Northern Arizona University, Flagstaff, AZ USA; 3 Department of Environmental and Occupational Health, Milken Institute School of Public Health, George Washington University, Washington, DC USA; 4 Minneapolis Veterans Affairs Health Care System, Minneapolis, MN USA; 5 University of Minnesota, Minneapolis, MN USA

**Keywords:** Bacterial infection, Antimicrobial resistance

## Abstract

**Background:**

Infections caused by antibiotic-resistant bacteria are increasingly frequent, burdening healthcare systems worldwide. As pathogens acquire resistance to all known antibiotics – i.e., become pan-resistant – treatment of the associated infections will become exceedingly difficult. We hypothesized that the emergence of pan-resistant bacterial pathogens will result in a sharp increase in human mortality.

**Methods:**

We tested this hypothesis by modeling the impact of a single hypothetical pan-resistant *Escherichia coli* strain on sepsis deaths in the United States. We used long-term data on sepsis incidence, mortality rates, strain dynamics, and treatment outcomes to parameterize a set of models encompassing a range of plausible future scenarios. All models accounted for historical and projected temporal changes in population size and age distribution.

**Results:**

The models suggest that sepsis deaths could increase 18- to 46-fold within 5 years of the emergence of a single pan-resistant *E. coli* strain. This large and rapid change contrasts sharply with the current expectation of gradual change under continuing multidrug-resistance.

**Conclusions:**

Failure to prevent the emergence of pan-resistance would have dire consequences for public health.

## Plain Language Summary

Antibiotic-resistant bacteria are an increasing risk to public health. As bacteria become resistant to all known antibiotics – i.e., become pan-resistant – treatment of infections will become extremely difficult. We hypothesized that the appearance of pan-resistant bacteria will result in a sharp increase in mortality. We tested this hypothesis using computer and mathematical modeling to see how a single hypothetical pan-resistant type of bacteria would impact deaths in the United States. Drawing from existing long-term data, deaths from infection in the general population could increase dramatically within 5 years of the emergence of a single pan-resistant type of common bacteria. Failing to prevent the emergence of pan-resistance would have dire consequences for public health.

## Abstract (web summary)

Koch et al. model scenarios of emergence of a single pan-resistant *Escherichia coli* strain in the United States. Findings suggest dire mortality outcomes and highlight the importance of measures to prevent the emergence of antimicrobial resistance.

## Introduction

Alexander Fleming was the first to discover an antibiotic and the first to warn about the risk of resistance to these drugs. With the specter of pan-resistant pathogens now on the horizon^1^, public health experts warn of an imminent post-antibiotic era – but Fleming’s pre-antibiotic generation was the last that could accurately envision a world without antibiotics. It is our central premise that treating pan-resistant infections will be considerably more difficult than treating extensively-resistant infections. Because of this, simple extrapolations from past trends will most likely underestimate the increase in disease burden and deaths that will accompany the emergence of pan-resistance^2,3^.

Although many bacterial infections are treated with antibiotics, few depend more on antibiotic treatment to ensure patient survival than does sepsis^4^. Sepsis is a major public health problem; an estimated 1.7 million cases and 350,000 sepsis deaths occur annually in the United States^5^. For these reasons, sepsis is ideal for modeling the real-world impact of pan-resistant bacterial pathogens.

Multidrug resistance – i.e., resistance to three or more classes of antibiotics – is increasingly common among sepsis-causing bacteria^6^. Despite this, sepsis case mortality has decreased over the past 40 years, likely due to improved diagnosis, better supportive care, and more aggressive antibiotic therapy^7,8^. This favorable trend could change abruptly as common sepsis-causing pathogens become pan-resistant.

*Escherichia coli* is one of the most common causes of sepsis, accounting for >15% of sepsis cases across all ages^6,9^. Multidrug resistance is increasing rapidly among human *E. coli* infections, driven largely by the expansion of a few extensively-resistant extraintestinal pathogenic *E. coli* strains^10–12^. In this study, we modeled how the emergence of novel pan-resistant extraintestinal pathogenic *E. coli* strains could impact sepsis mortality. We focused on predicting the magnitude of mortality change rather than the exact timing of emergence, which carries large uncertainty. Our results demonstrate that a single pan-resistant *E. coli* strain, with an emergence pattern similar to a recently emerged multidrug-resistant strain, ST131-*H*30R, could rapidly cause a greater than 10-fold rise in sepsis deaths compared to current mortality trends under multidrug-resistance.

## Methods

### Sepsis incidence and mortality

We used published data from hospital discharge survey data, electronic health records, and insurance claims^7,13–15^ to estimate sepsis incidence and mortality (Supplementary Figs. S1 and S2). Patient consent was not required as all data were publicly available and de-identified. No ethical approval from overseeing institutional bodies was sought because it was not required for this study. Claims-based data underestimate true sepsis incidence due to variable diagnosis and coding practices^13^. Therefore, we derived a correction factor for claims-based estimates from 2009 to 2012, when data were available from both sources. We calculated the mean difference in total U.S. sepsis cases for the electronic health records from Rhee et al.^13^ and the claims-based data of Stoller et al.^15^. We applied this correction factor (401,671 cases) to all claims-based incidence estimates (Supplementary Fig. S3).

Similarly, we used claims-based data and data from electronic health records to estimate sepsis mortality rates – rather than death-certificate-based data (e.g., CDC (2023)^16^) – which are likely to underestimate sepsis mortality because they may not specify sepsis or organ dysfunction as a contributing factor to death, even when those conditions are present^8,17^. Sepsis mortality rates required no corrections for variable diagnosis and coding practices.

### Accounting for population size, age, and demographic trends

We modeled sepsis incidence and case mortality rate separately for four age classes (<15 years, 15–44 years, 45–64 years, ≥65 years). We also accounted for actual changes in U.S. population size and age distribution since 1975 and for projected changes after 2019 (Supplementary Figs. S4 and  S5). To establish the age distribution of sepsis incidence, we extracted age-stratified sepsis incidence data from 11 years of U.S. National Hospital Discharge Survey reports (1990–2000)^18^ by following the methodology of Martin et al.^14^. Age class-specific incidence did not vary over that 11-year period; therefore, we used a constant mean age distribution of sepsis incidence over all years of the model (1975–2050; Supplementary Fig. S6). Similarly, we assumed a fixed age distribution of sepsis case mortality rates for all model years, using age class-specific mortality rates from Rhee et al.^13^ for the year 2014 (Supplementary Fig. S7). We translated those published age class-specific case mortality rates into the four age classes used in our model using a linear mixing model of one-year age classes and assuming a maximum age of 99 years (<15 years: 0.029 deaths per sepsis case, 15–44 years: 0.128, 45–64 years: 0.135, ≥65 years: 0.297). We used U.S. Census Bureau data^19^ to calculate age class-specific population sizes for each year of the model through 2019, and U.S. Census Bureau projections^20^ to estimate age class-specific population sizes for model years 2020 and later.

To assess the trend of sepsis incidence over time – independent of the effects of changing population size and age distribution – we used the age class-specific incidence and population estimates derived above to calculate annual population-adjusted sepsis incidence, standardized to the observed age distribution in 1979, the first year of our observed time-series data (Supplementary Fig. S1).

We then fit models to the population- and age-adjusted data, to encompass a range of future scenarios projected to 2050: (A) most conservative: logistic growth assuming annual sepsis incidence plateaus at the most recent observed maximum, (B) moderately conservative: logistic growth assuming annual sepsis incidence plateaus at two times the most recent observed maximum, and (C) least conservative: two-part linear growth for the years 1975–2000 and for 2000–2050 (Supplementary Fig. S1).

To back-transform the projected population- and age-adjusted sepsis incidence values to unadjusted raw incidence values, we first interpolated total U.S. population size and population size within each of the four age classes for all time points in the model (1975–2050) using a smoothing spline (degrees of freedom = 2, smoothing parameter = 0 for all). We then calculated the unadjusted raw sepsis incidence values (for each of the three potential future scenarios) by multiplying the scenario’s time-series vector of modeled population- and age-adjusted sepsis incidence values by the time-series vector of U.S. population size and dividing by 100,000. Next, for each age class, we multiplied the resulting scenario’s time-series vector by the age-specific sepsis incidence value (fixed for all modeled time points) and the time-series vector of the proportion of total U.S. population in that age class and divided by the proportion of the 1979 U.S. population in that age class. The sum across all four age classes of the resulting time-series vectors yielded the unadjusted raw sepsis incidence values for each scenario.

To assess the trend of sepsis mortality over time – independent of the effects of the population’s changing age distribution – we used the age class-specific mortalities and population estimates derived above to calculate annual age-adjusted sepsis mortality standardized to the observed age distribution in 1979 (Supplementary Fig. S2). We fit a model to these age-adjusted data using a smoothing spline (degrees of freedom = 7, smoothing parameter = 1) to derive a time-series vector of age-adjusted sepsis mortalities for all time points from 1975 to 2050. We then back-transformed the projected age-adjusted sepsis mortality values to unadjusted raw mortality values, using steps similar to those described above for incidence. The sum of the products of unadjusted raw incidence and mortality values for each age class produced an estimate of the total number of U.S. sepsis deaths from 1975 to 2050 for each of the three future scenarios modeled (Supplementary Fig. S8).

### Modeling pan-resistance

We constructed three models of sepsis incidence to account for the uncertainty of future sepsis trends (Supplementary Fig. S1). The most conservative model scenario assumed sepsis incidence will plateau at the most recent observed maximum and was fit by ordinary least-squares using a logistic function. The moderately conservative model scenario assumed sepsis incidence will plateau at twice the most recent observed maximum and was fit by ordinary least-squares using a logistic function. The least conservative model scenario assumed sepsis incidence will continue to increase linearly at the same rate that it has increased since 2000. We fit two separate linear models for the periods 1979–2000 and 2000–2014 using ordinary least-squares to represent the least conservative model scenario.

All models spanned the time period from 1975 to 2050 and had time steps of 0.01 years. There were two *E. coli* strains represented in all models: (1) multidrug-resistant *E. coli* ST131-*H*30R and (2) a hypothetical pan-resistant *E. coli* strain with the same prevalence dynamics at emergence as were observed for ST131-*H*30R. We assumed that *E. coli* ST131-*H*30R first emerged in the year 2000^21^. The year of emergence (2040) of the pan-resistant *E. coli* strain was estimated as the median of the distribution of emergence times generated from 1000 simulations. Simulations began in the year 2024 and assumed a 0.04% probability of pan-resistance emergence at each time step of the model^22^. The prevalences of the two *E. coli* strains (ST131-*H*30R and pan-resistant strain) did not vary among models.

To bracket the uncertainty of mortality risk associated with a pan-resistant *E. coli* strain, we used two mortality rates for our hypothetical pan-resistant strain. We based these two mortality rates on clinical data showing increases in mortality with hourly delays in broad-spectrum antibiotic administration. For the higher mortality rate, we used data from Kumar et al.^23^ for the fraction of patients dying after a 24-to-36-h delay in antibiotic treatment. For the lower, more conservative mortality rate, we used data from Seymour et al.^24^, and extrapolated the trend in mortality to the same 24-to-36-h treatment delay. Both of these mortality rates were derived from sample populations that roughly corresponded to the oldest age class in our model (≥65 years). Therefore, we calculated the fold-increase in mortality for the ≥65 years age class over the non-pan-resistant modeled mortality for 1996 for the higher mortality estimate and for the year 2015 for the lower estimate. The years 1996 and 2015 correspond to mean dates of data collection for studies^23^ and^24^. We then applied that fold-change (+1.9-fold for the lower mortality estimate and +4.8-fold for the higher mortality estimate) to each of the sepsis mortality rate time-series vectors for each age class for the period subsequent to the emergence of pan-resistance.

### Reporting summary

Further information on research design is available in the Nature Portfolio Reporting Summary linked to this article.

## Results

### Sepsis incidence and mortality

**Fig. 1:**
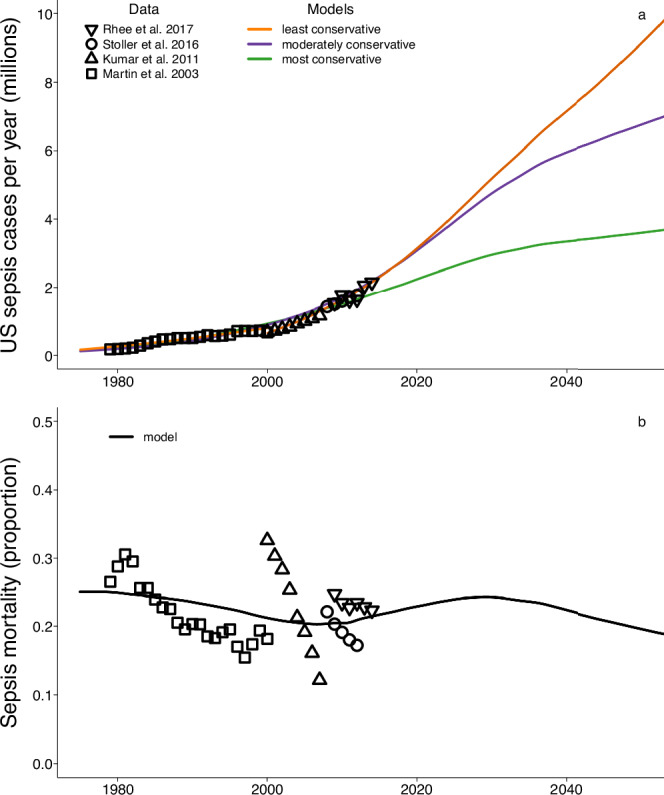
Historic and projected time trends for sepsis incidence and sepsis case mortality rate for the baseline case without pan-resistance. The estimated number of sepsis cases per year (**a**) from all causative agents in the United States has increased since 1979, whereas the estimated sepsis mortality rate has declined (**b**). Annual estimates (symbols) are based on hospital discharge survey data, electronic health records, and insurance claims^7,13–15^. The claims-based sepsis case data of Stoller et al.^15^ and Kumar et al.^7^ were adjusted to correct for variable diagnosis and coding practices (see Methods). We predicted sepsis incidence through the year 2050, with three models (colored lines) that represented a range in possible future scenarios for sepsis incidence (Supplementary Fig. S1). The models also accounted for projected changes in population size and population age structure. We also predicted U.S. sepsis mortality rates through the year 2050 (black line) using a model of age-adjusted mortality (Supplementary Fig. S2) and accounting for projected demographic shifts in the U.S. population (Supplementary Figs. S4 and S5).

To define the recent trajectory of sepsis cases and mortality in the U.S., we collated incidence estimates from the peer-reviewed literature. Studies spanned from 1979 to 2014 and included approximately 100 million hospitalizations. Estimates of sepsis incidence were derived from hospital discharge survey data, electronic health records, and insurance claims^7,13–15^. Over this time period, medical record coding practices for sepsis have varied, an artifact that may contribute to observed temporal trends^25^ (Supplementary Fig. S3). Nonetheless, these data showed a concurrent increase in sepsis incidence and decrease in sepsis case mortality rate from 1979 to 2014 (Fig. 1).

To predict sepsis incidence under the hypothetical baseline scenario that no major *E. coli* strains transition to a pan-resistant phenotype, regression models were fit to the historical data and extrapolated to the year 2050. We fit three models to encompass a range of future scenarios (Fig. 1A, Supplementary Fig. S1). The most conservative was a logistic growth model that assumed annual sepsis incidence would increase and then plateau at the most recent observed maximum. A moderately conservative logistic regression model assumed incidence would plateau at two times the most recent observed maximum. For the least conservative model, we fit a two-part linear regression to the observed data; for the years 1979–2000, and for the years 2000–2014. For all models, we accounted for differences in population size and population age structure through time, following the steps detailed in the Methods.

The most conservative model (Fig. 1A, green line) projected that future sepsis incidence would increase gradually to about 3.6 million cases per year in 2050, consistent with the hypothesis that recent sepsis trends may be driven more by changing coding practices than by true changes in incidence^25^. The moderately conservative model (Fig. 1A, purple line) projected that incidence would rise to approximately 6.8 million cases per year by 2050. The least conservative model (Fig. 1A, orange line) projected that population- and age-adjusted incidence would increase yearly by 23.5 cases per 100,000 (Supplementary Fig. S1) and reach roughly 9.1 million cases per year by 2050. All models fit the observed data similarly using the Akaike information criterion (Supplementary Fig. S1) and provide a framework necessary to evaluate potential changes in sepsis mortality.

To predict the number of U.S. sepsis deaths, assuming no pan-resistant *E. coli* strains, we applied the three sepsis incidence models described above to estimates of sepsis mortality rate (Fig. 1B). First, we fit a smoothing spline to existing mortality rate data^7,13–15^ and extrapolated that model to the year 2050, accounting for projected changes in population size and population age structure (Supplementary Fig. S2). Next, we used the three scenarios to predict future sepsis deaths in the U.S. by multiplying projections of incidence and mortality rate (Supplementary Fig. S8). The resulting rise in the number of sepsis deaths largely mirrored the three incidence patterns, with attenuated trajectories in later years due to the projected long-term decline in case mortality rate.

### Pan-resistance

Next, to model the emergence of a theoretical pan-resistant *E. coli* strain, we used the natural history of a well-described multidrug-resistant *E. coli* strain: the *H*30R subclone of multi-locus sequence type 131 (ST131-*H*30R). The biological basis for ST131-*H*30R’s rapid success is still under investigation, but has been proposed to include the acquisition of fluoroquinolone resistance-determining mutations and the CTX-M-15 beta-lactamase gene during a time of high prescription rates for fluoroquinolones and extended-spectrum cephalosporins^26,27^. Concerningly, *H*30R has recently acquired carbapenemase-mediated resistance to carbapenems, a traditional last defense against extensively-resistant *E. coli* strains, and is now a – and, in some locales, the – leading carbapenem-resistant *E. coli* lineage^28–31^. Additional likely contributors to ST131-*H*30R’s dramatic expansion include its acquisition of multiple fitness-conferring accessory genes^26,32^, distinctive methylation patterns^33^, and mutations in regulatory regions^32,34^ that may enhance its virulence, commensalism, and/or transmission ability, phenotypes for which ST131-*H*30R is well known^28,34–38^.

**Fig. 2:**
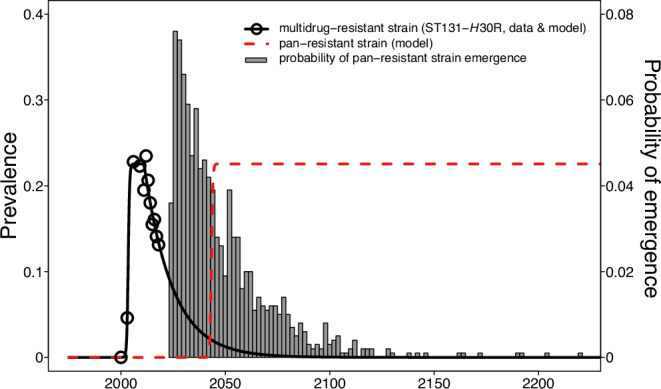
Time course of emergence and prevalence of ST131-*H*30R and a hypothetical pan-resistant *Escherichia coli* strain. The prevalence of ST131-*H*30R, a multidrug-resistant *E. coli* strain (black circles), increased rapidly following first detection in 2003 before gradually declining since 2011^21,39^. We fit a model (black line) to these data, describing a logistic increase in ST131-*H*30R prevalence (*N*_0_ = 8.45 × 10^−4^%, *r* = 2.94% per year, *K* = 22.6%), followed by an exponential decline (*N*_0_ = 22.6%, *r* = −7.44% per year). We used data from Carter et al.^22^ to simulate the date of emergence (*n* = 1000, gray histogram) of a pan-resistant *E. coli* strain. Using the median date of simulated emergence (2040) along with the parameters describing the logistic increase of ST131-*H*30R in the early 2000’s, we created a model (red dashed line) describing the predicted prevalence of a hypothetical pan-resistant *E. coli* strain.

To characterize the dynamics of this multidrug resistant *E. coli* strain, we fit a logistic growth model to the ST131-*H*30R prevalence data from 2000 to 2009 and extrapolated to 2011, when ST131-*H*30R began to decline. We then fit an exponential function to the observed ST131-*H*30R prevalence from 2011 to 2018^39^ and extrapolated to the year 2050 (Fig. 2).

To model the timing for the emergence of a pan-resistant *E. coli* strain in the absence of new antibiotics coming to market, we assigned a probability of acquiring pan-resistance for each time-step of our model (1/100th of a year) and ran 1000 iterations to find the median date of first appearance. We assumed strains had a 20% probability of becoming pan-resistant within 5 years^22^, and thus a 0.04% probability at each time-step of our model, starting in the year 2024. Under these assumptions, our model predicted the appearance of a pan-resistant *E. coli* strain in 2040 (Fig. 2, red dashed line; 90% CI: 2025–2094, gray columns).

We hypothesized that a pan-resistant *E. coli* strain would emerge similarly to ST131-*H*30R, with a rapid rise to prominence followed by a tapering off to a relatively constant prevalence. Although ST131-*H*30R has recently decreased in prevalence, the concurrent rise of a new multidrug resistant *E. coli* strain, ST1193, has offset the decline, such that the net sum of their prevalence is 20% of clinical *E. coli* isolates in the United States^21,39^ (Supplementary Fig. S9). We therefore modeled the dynamics of the hypothetical pan-resistant strain according to the logistic function defined by the earlier appearance of ST131-*H*30R (Fig. 2). Specifically, for each of the 1000 model iterations, we set the initial prevalence (*N*_0_) of the pan-resistant strain to 0.01% at the moment of appearance and assumed a growth rate (*r* = 2.94% per year) and carrying capacity (*K* = 22.6%) equivalent to those of the model fit to the observed ST131-*H*30R data, above.

The expected mortality risk associated with pan-resistant *E. coli* is uncertain, so to bracket this uncertainty with both a low and a high mortality estimate we used data from studies showing increasing mortality with hourly delays in broad-spectrum antibiotic administration^23,24^. As detailed in the Methods, we applied these estimates to the time-series of age class-specific mortality rates derived for sepsis patients infected with *E. coli* ST131-*H*30R to estimate age-specific mortality rates associated with the pan-resistant *E. coli* strain after its emergence in 2040 (Supplementary Fig. S10). Under these assumptions, mortality rates for infections due to the hypothetical pan-resistant strain ranged from 34% to 96% for the ≥65 years age class, a substantial increase over current sepsis mortality rates, which include all causative agents (not just *E. coli*).

We modeled the number of U.S. deaths (*D*_*i*_) expected in each time-step (*i*) from both the current multidrug resistant *E. coli* ST131-*H*30R strain and the hypothetical pan-resistant *E. coli* strain as:

$${D}_{i}=0.16 \, {{\cdot }} \, {\sum}_{j}^{n=4}{S}_{{ij}} \, {{\cdot }} \, ({M}_{{{{\rm{pre}}}}{ij}} \, {{\cdot }} \, {P}_{131i}+{M}_{{{{\rm{post}}}}{ij}} \, {{\cdot }} \, {P}_{{{{\rm{pan}}}}i})$$

**Fig. 3:**
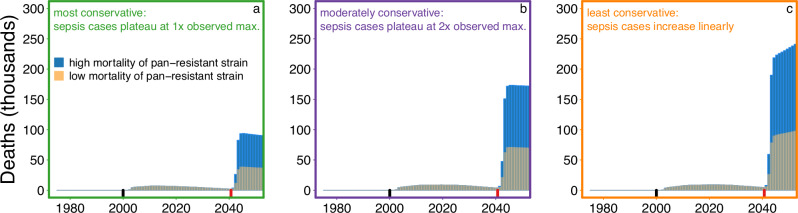
Temporal trends in annual deaths due to sepsis in the United States from ST131-*H*30R and a hypothetical pan-resistant *E. coli* strain. Annual deaths due to sepsis increased over time under all three scenarios of projected sepsis incidence from Fig. 1: **a** the most conservative scenario, in which population- and age-adjusted sepsis incidence levels off at the most recent observed maximum (Supplementary Fig. S1); **b** the moderately conservative scenario, in which population- and age-adjusted sepsis incidence levels off at twice the most recent observed maximum (Supplementary Fig. S1); and **c** the least conservative scenario, in which population- and age-adjusted sepsis incidence continues to increase linearly (Supplementary Fig. S1). Two sepsis mortality rates for a pan-resistant strain were modeled to yield the total predicted annual deaths due to sepsis shown in the gray versus blue bars. Under all scenarios, the predicted number of sepsis deaths increased dramatically with the modeled emergence in 2040 of the pan-resistant *E. coli* strain. The year of first appearance for ST131-*H*30R and for the pan-resistant *E. coli* strain are indicated by the small black and red lines, respectively.

Here, 0.16 is the estimated proportion of sepsis cases attributable to *E. coli*^6,40^, *S*_*ij*_ is the total number of projected U.S. sepsis cases at time *i* for age class *j*, *M*_pre*ij*_ is the projected sepsis mortality rate for all susceptible and multidrug resistant *E. coli* at time *i* for age class *j*, *M*_post*ij*_ is the projected sepsis mortality rate for the pan-resistant strain at time *i* for age class *j*, and *P*_131*i*_ and *P*_pan*i*_ are the projected prevalences at time *i* for the ST131-*H*30R strain and the pan-resistant strain, respectively. Projections for all variables used time-steps of 0.01 years, which were subsequently summed to yield annual model estimates of U.S. deaths. Our model spanned the years 1975–2050. As described above, we used three scenarios of projected sepsis cases over time and two mortality rate scenarios for the pan-resistant strain, resulting in six separate time-series projections of sepsis deaths attributable to the ST131-*H*30R strain and a pan-resistant strain (Fig. 3).

## Discussion

All variations of the model suggested a precipitous increase in annual deaths due to sepsis with the emergence of a pan-resistant *E. coli* strain in 2040 (Fig. 3). Even with the most conservative estimates, our models suggest that the emergence of a single pan-resistant strain with the relative success of *E. coli* ST131-*H*30R would increase *E. coli* sepsis deaths 18-fold (by an average of 7240 per year) within five years. These data indicate that failing to prevent extraintestinal pathogenic *E. coli* strains from transitioning to a pan-resistant phenotype would have dire consequences for public health and clinical medicine.

We initiated this project with the assumption that most people in high-income countries lack the perspective needed to fathom a world with pan-resistant bacterial pathogens, and therefore likely underestimate the potential risks posed by the emergence of such organisms. Our findings indicate that sepsis deaths could rise rapidly to ominous levels once even the most aggressive antibiotic therapies are rendered ineffective. Although sepsis survival rates have improved over the last 40 years, hourly delays in antibiotic treatment are accompanied by progressive increases in sepsis mortality^23,24^. While such studies have received criticism^41–43^, with pan-resistance, no effective antibiotics are available, so treatment delays become infinite. In such a world, treatment outcomes will be strikingly worse for patients infected with pan-resistant – as opposed to merely extensively-resistant – pathogens.

Under the assumptions of our models, the predicted impact of pan-resistant *E. coli* strains on sepsis deaths is extreme. Yet even our worst-case model ignores the likelihood that a pan-resistant *E. coli* strain will likely accelerate the current rate of increase in sepsis incidence. Currently, 2–5% of untreated cystitis cases progress to sepsis^44–46^. Without effective antibiotics, many more bladder infections will go essentially untreated, presumably increasing sepsis incidence and further elevating the rapid increase in sepsis deaths predicted by our models.

Paradoxically, even as sepsis incidence has risen in the U.S., sepsis case mortality rate has declined. Improvements in sepsis awareness, detection, and management may all contribute to this pattern. For instance, efforts to detect sepsis earlier in its course, in milder forms, and in patients with other primary acute conditions have contributed to the apparent rise in sepsis incidence^13^. Other suspected contributors to rising sepsis rates include increasing rates of invasive procedures, growing numbers of immune-suppressed individuals, and emergence and expansion of more virulent *E. coli* lineages^25,47^. At the same time, increased sepsis awareness, early detection, and improved treatment regimens have decreased sepsis case mortality rates^8,48–50^. However, our findings suggest that the emergence of pan-resistant *E. coli* strains could undermine these advances. Specifically, we predict a sharp rise in sepsis deaths if pan-resistant *E. coli* infections eliminate the option of appropriate antibiotic therapy.

New antibiotics are needed to head off the emergence of pan-resistant bacterial pathogens, but will only delay, not prevent, such emergence, unless several underlying issues are addressed. First, we must reduce the forces that currently promote the evolution and dissemination of antibiotic-resistant pathogens. Globally, this means improving antibiotic stewardship in human and veterinary medicine and in food-animal production. Indeed, abundant evidence suggests that resistant organisms and mobile resistance determinants from antibiotic-exposed animals are transmitted to humans via multiple routes and can adversely impact human health^51–53^. In high-income countries, human and animal antimicrobial stewardship efforts could benefit if rapid and novel diagnostics for pathogen detection and antimicrobial susceptibility determination were integrated better into standard clinical protocols. In lower- and middle-income countries, preventing dissemination of resistant pathogens also requires providing the infrastructure necessary for clean drinking water and hygienic sewage management. Second, we must solve the market failures that have driven drug companies away from antibiotic development and that fuel antibiotic overuse^50^. Of the multiple proposed solutions, the most promising are cash rewards that link payment to conservation and access^54^. Third, we must view antibiotic resistance as a persistent, likely permanent problem (one which some authorities regard as being even more threatening in the near term than climate change)^55^, which means that we must develop new antibiotics in a thoughtful, coordinated manner. Until all these issues are addressed, each new antibiotic will only delay the sharp increase in deaths that our models predict will accompany the emergence of pan-resistant pathogens.

Multiple microbial species contribute to the global antibiotic resistance threat. Our findings, based on just a single *E. coli* strain in the U.S., provide a data-driven, yet incomplete picture of this looming global crisis. Similar modeling exercises for other major pathogens could give clinicians, policymakers, and the general public a more comprehensive preview of a post-antibiotic world, so they can accurately prioritize antibiotic resistance in relation to the many other current public health challenges.

## Supplementary information

Supplementary Information

Reporting Summary

## Data Availability

All source data are available online^56^.

## Code availability

Code for implementing all models and analyses, and for generating all figures is available online^56^.
