# Supplementary material for: Predicting sepsis mortality into an era of pandrug-resistant E. coli through modeling
Source: Commun Med (Lond). 2024 Dec 26;4:278. doi: 10.1038/s43856-024-00693-7 (PMC11671531; doi:10.1038/s43856-024-00693-7)
Supplement: Supplementary file 1 — Supplementary Information [file 43856_2024_693_MOESM1_ESM.pdf]

communications medicine

Supplementary Information for

Predicting sepsis mortality into an era of pandrug-resistant *E. coli* through modeling

Benjamin J. Koch, Daniel E. Park, Bruce A. Hungate, Cindy M. Liu, James R. Johnson, Lance B. Price

Correspondence to: ben.koch@nau.edu

This PDF file includes:

Supplementary Figures: Figs. S1 to S10

Note:

Cited references in this file are indicated by the same numbers listed in the *References* section of the main text.

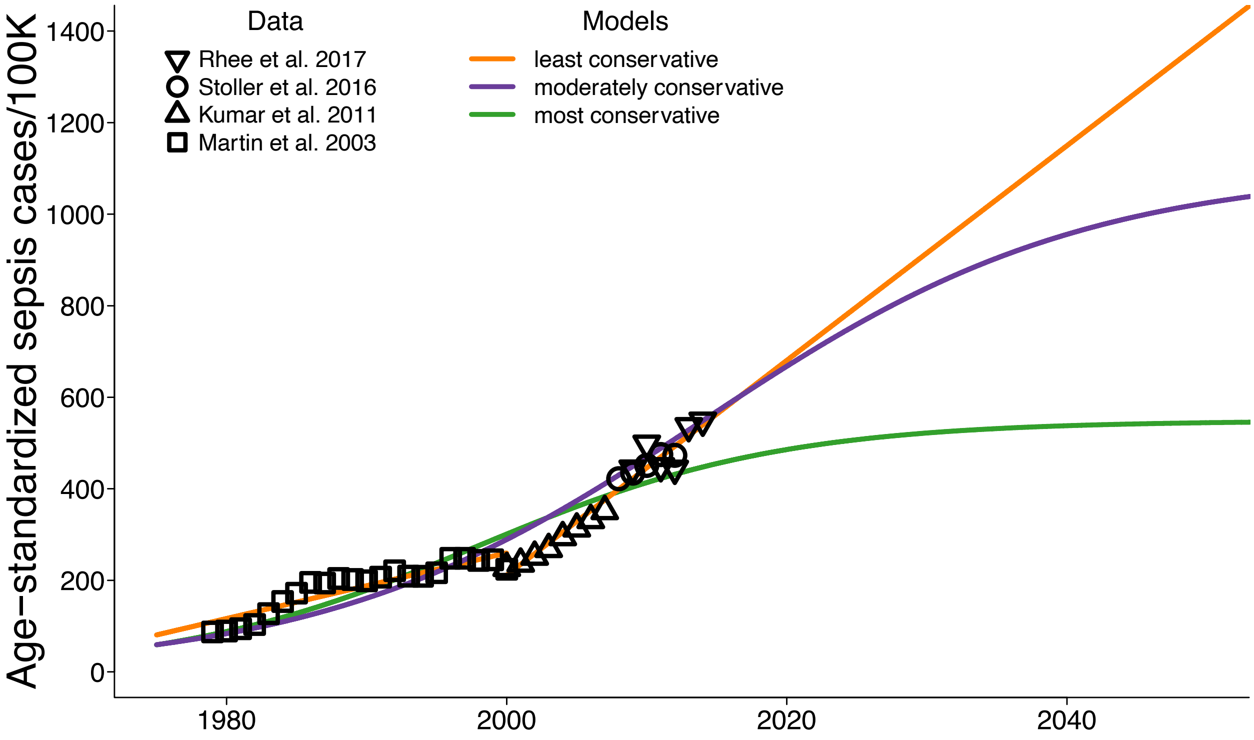

Figure S1. Historic and projected time trends for population- and age-adjusted sepsis incidence. Sepsis incidence from all causative agents in the United States, adjusted for changes in population size and population age structure has increased since 1979, indicating that population and demographic trends do not account for the observed rise in U.S. sepsis cases. All sepsis incidence estimates are standardized to the U.S. population age distribution in 1979 (Fig. S5). We predicted population- and age-adjusted sepsis incidence through the year 2050, by fitting three models (colored lines) to the observed data[7,13–15], choosing models that represented a range in possible future scenarios for sepsis incidence. All models fit the data comparably well (most conservative model parameters: x_0 = 1998, L = 549 cases per 100,000 population, k = 0.092 per year, Akaike information criterion (AIC) = 438; moderately conservative model parameters x_0 = 2014, L = 1098 cases per 100,000 population, k = 0.073 per year, AIC = 427; least conservative model parameters from 1979 to 2000 of $a = -1.41\times10^4$ cases per 100,000 population, b = 7.17 cases per 100,000 population per year and parameters from 2000 to 2014 of $a = -4.67\times10^4$ cases per 100,000 population, b = 23.5 cases per 100,000 population per year, AIC = 398). The claims-based sepsis case data of Stoller et al. (2016)[15] and Kumar et al. (2011)[7] were adjusted to correct for variable diagnosis and coding practices (see Fig. S3).

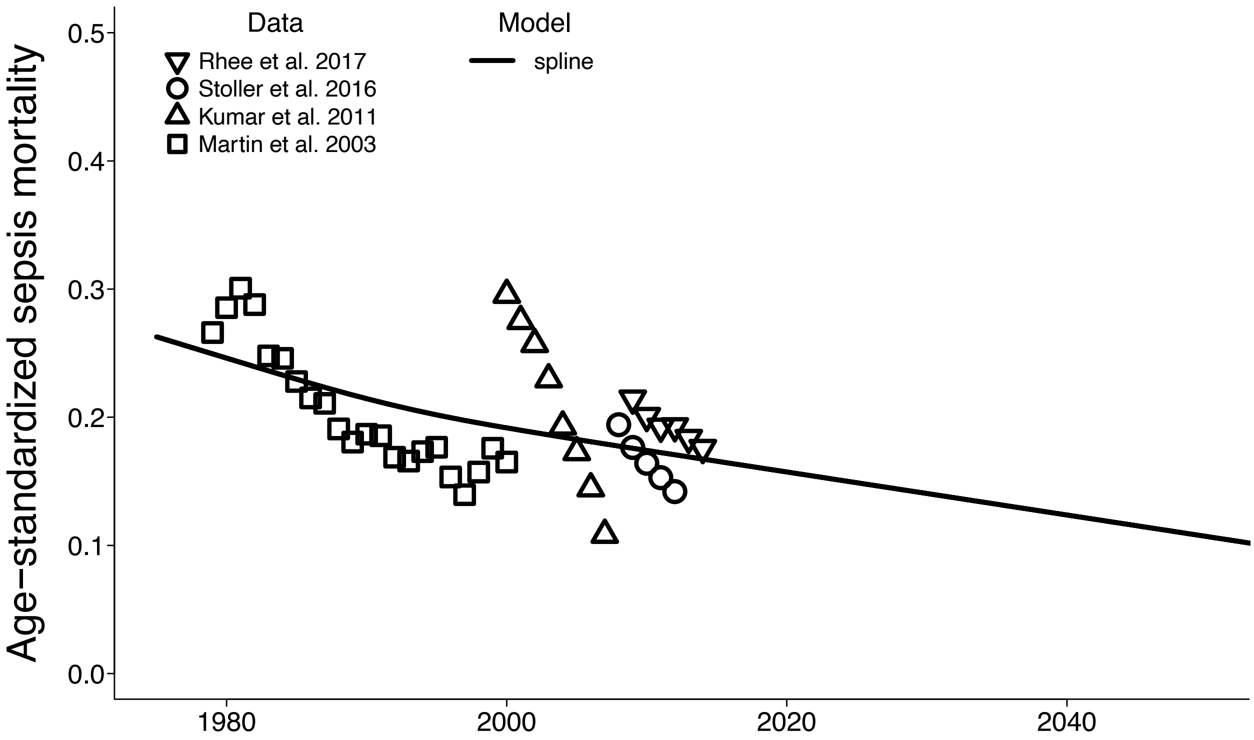

Figure S2. Historic and projected time trend for age-adjusted sepsis case mortality rate. The estimated U.S. sepsis mortality rate from all causative agents has declined since 1979, indicating that sepsis mortality is decreasing in spite of the demographic trend toward an older U.S. population. Annual estimates (symbols) are based on hospital discharge survey data, electronic health records, and insurance claims[7,13–15]. All sepsis mortality estimates are standardized to the U.S. population age distribution in 1979 (Fig. S5). We predicted age-standardized U.S. sepsis mortality rates through the year 2050 using a smoothing spline (black line, degrees of freedom = 7, smoothing parameter = 1).

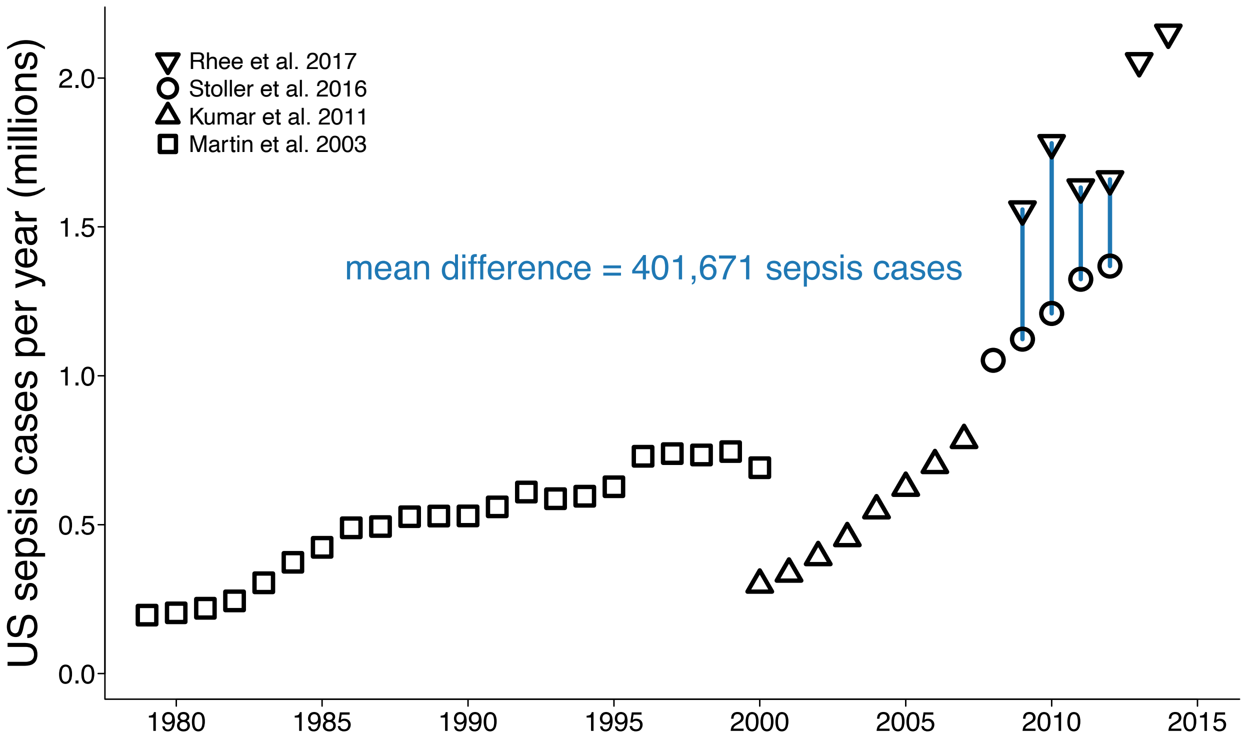

Figure S3. Estimated annual number of sepsis cases in the United States since 1979. Data were compiled from four different studies[7,13–15], two of which (Kumar et al. 2011 and Stoller et al. 2016)[7,15] were based on insurance claims data that are thought to underestimate sepsis incidence due to variable diagnosis and coding practices. To correct for this underestimate, we calculated the mean difference in sepsis cases for the four years of data where both claims-based data and electronic health record-based data were available. We then added this correction factor to all 13 claims-based annual estimates.

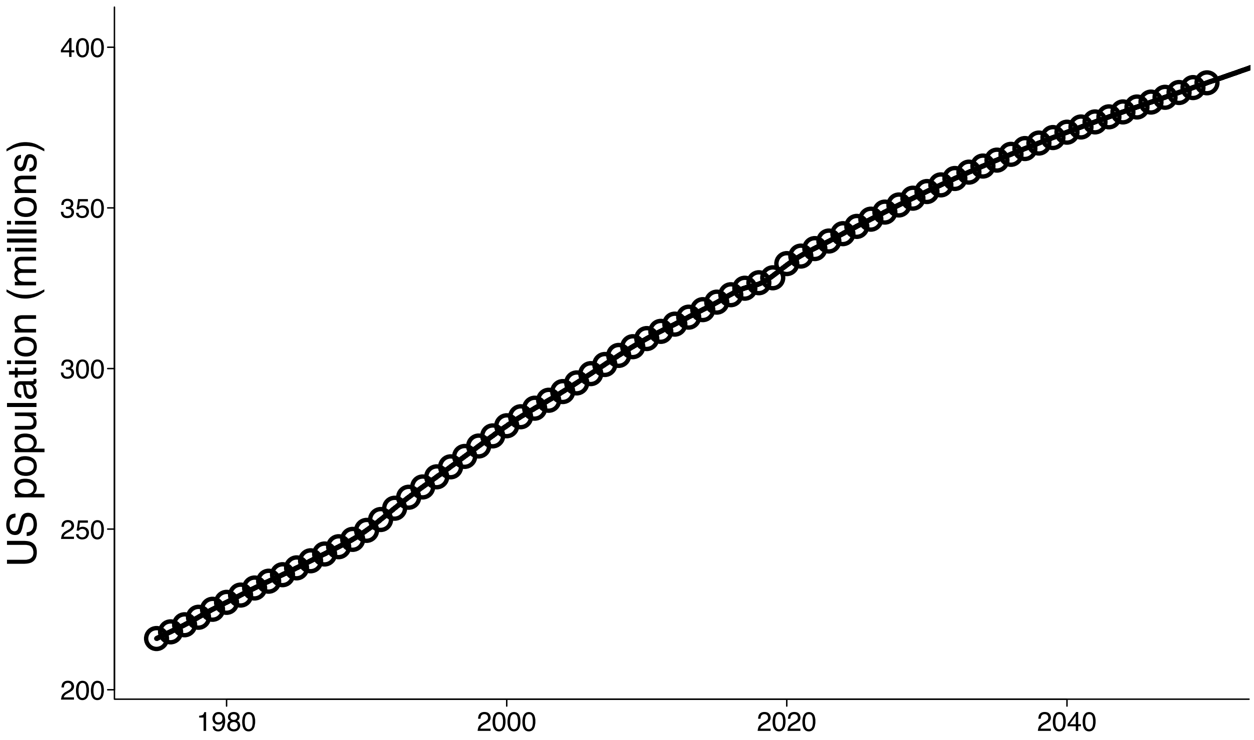

Figure S4. U.S. population estimates for each year of the model (1975-2050). Data (1975-2019) and projections (2020-2050) are from the U.S. Census Bureau[19,20]. Black line is a smoothing spline (degrees of freedom = 2, smoothing parameter = 0).

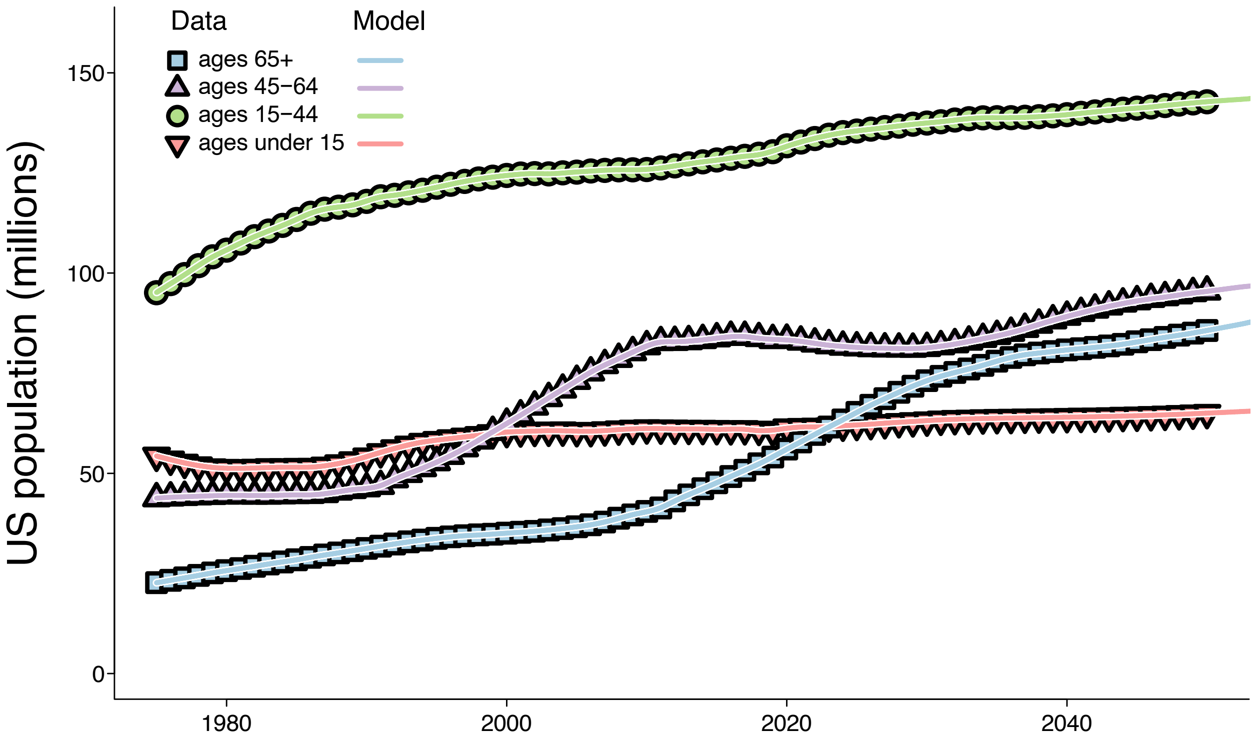

Figure S5. Age class-specific U.S. population estimates for each year of the model (1975-2050). Data (1975-2019) and projections (2020-2050) are from the U.S. Census Bureau[19,20]. Lines are smoothing splines (degrees of freedom = 2, smoothing parameter = 0 for all).

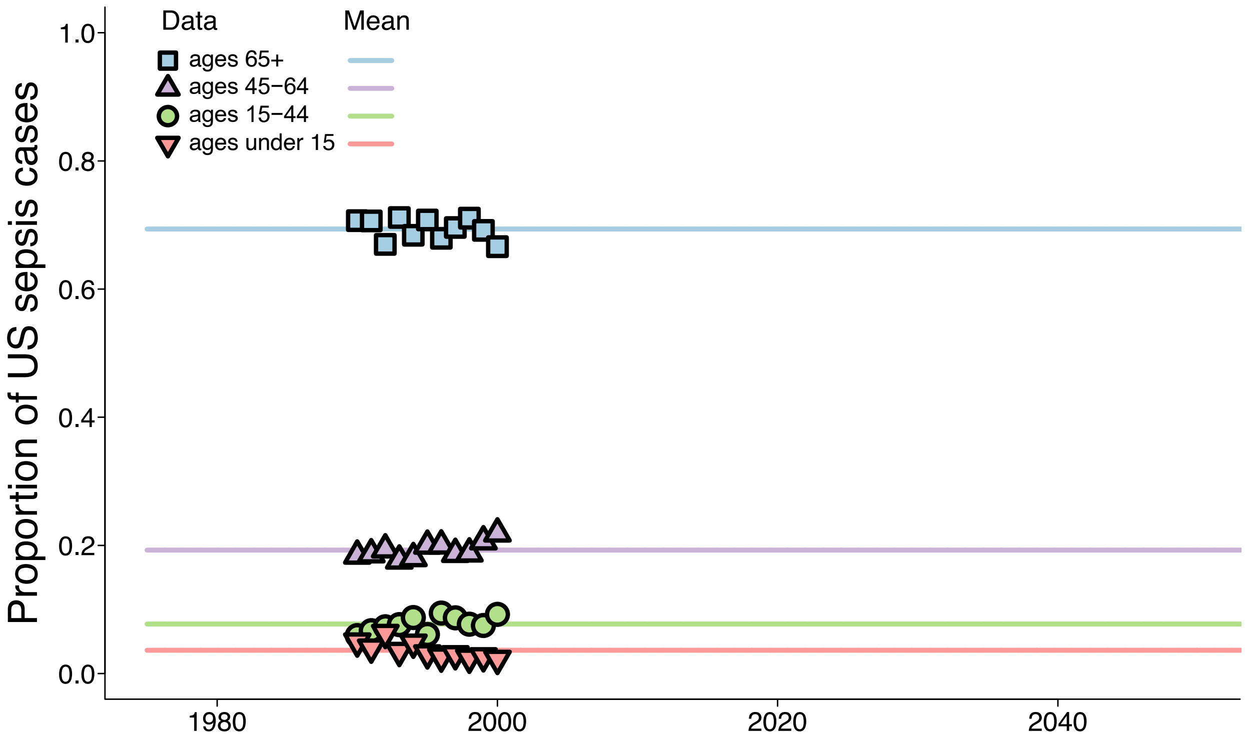

Figure S6. Age class-specific sepsis incidence as a proportion of total U.S. sepsis incidence. Data were compiled from the U.S. National Hospital Discharge Survey[18]. Age class-specific sepsis incidence did not vary over the 11-year period for which data were available. Colored lines indicate mean values for each age class; which we used at all time steps of the model (1975-2050).

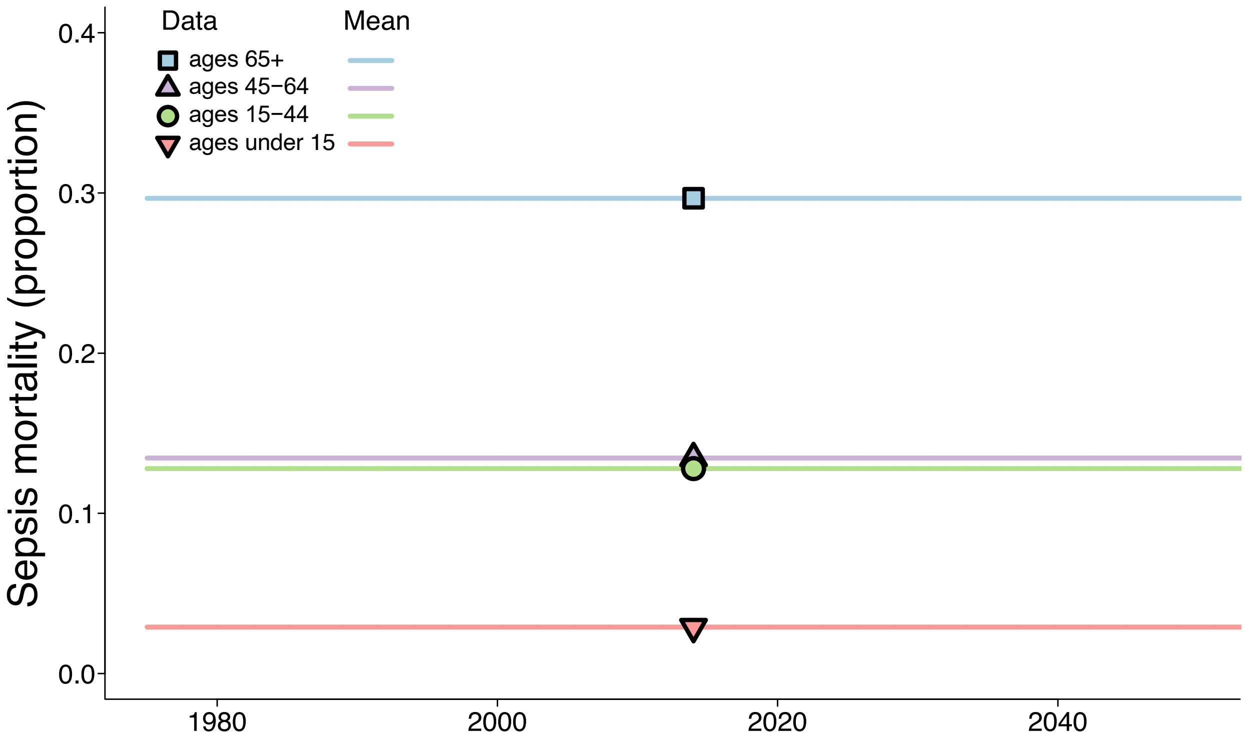

Figure S7. Sepsis case mortality rates for each age class in model. Estimates[13] were available only for 2014 and the value for each age class was used at all time steps of the model (colored lines, 1975-2050).

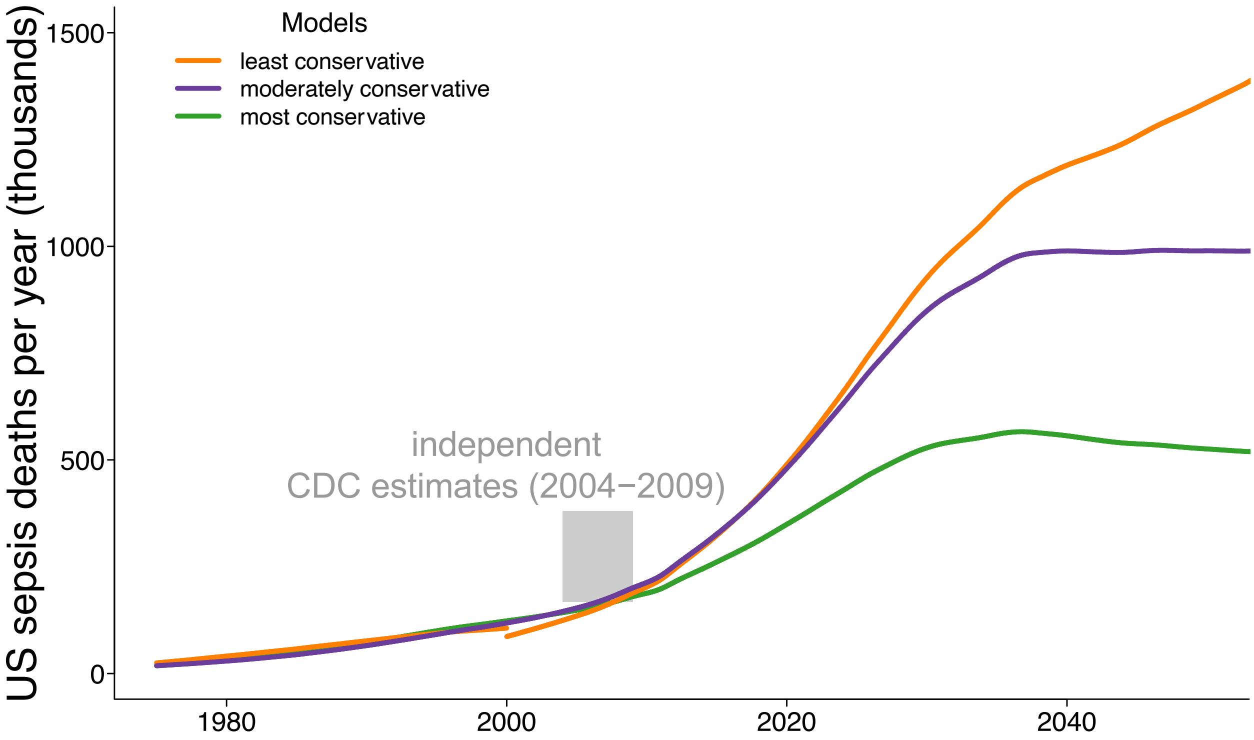

Figure S8. Modeled sepsis deaths in the United States from all causative agents, assuming no emergence of a pan-resistant bacterial strain. The three scenarios correspond to the three scenarios of future sepsis incidence (see Fig. 1A and Fig. S1), and all three incorporate the modeled long-term decline in sepsis mortality rate (Fig. S2), as well as projected changes in U.S. population size (Fig. S4) and population age structure (Fig. S5). The gray box delimits the range in U.S. sepsis deaths estimated by the U.S. Centers for Disease Control and Prevention[17] for the period 2004 to 2009.

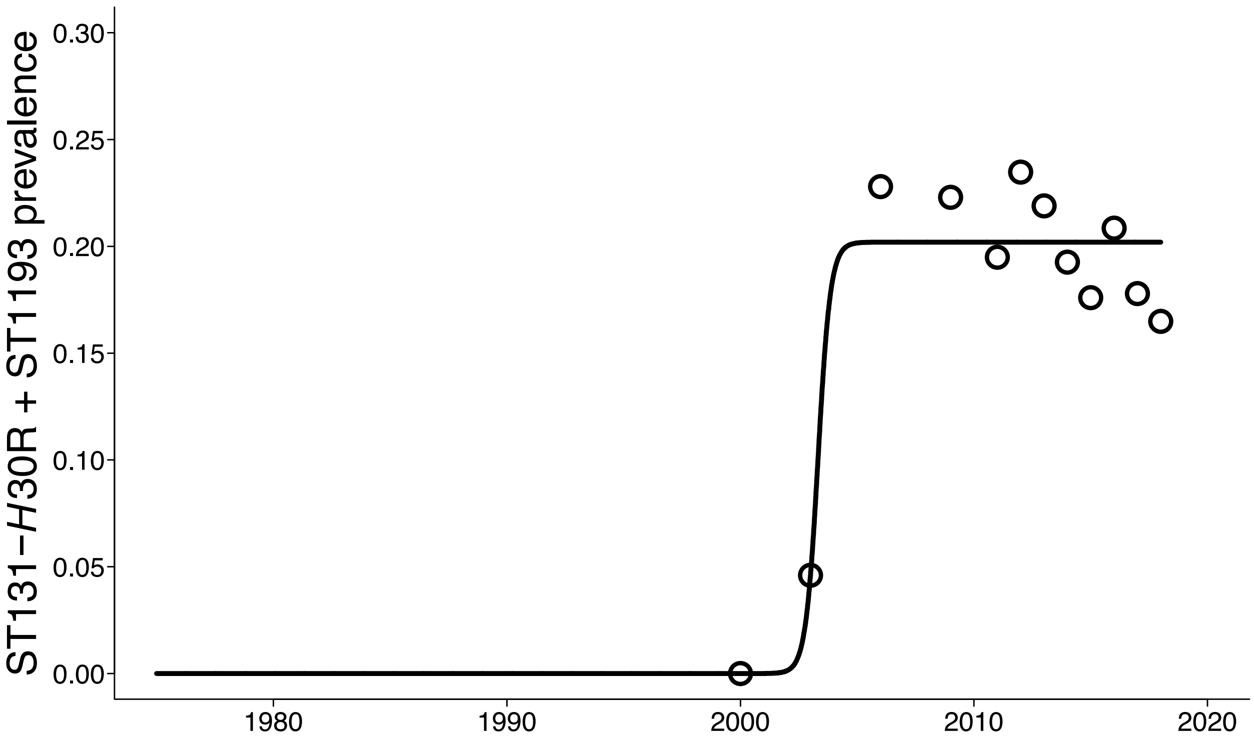

Figure S9. Combined prevalence of ST131-*H*30R and ST1193. The combined prevalence of two multidrug-resistant extraintestinal pathogenic *Escherichia coli* strains, ST131-*H*30R and ST1193, increased rapidly following first detection in 2003 and have remained at approximately 20% of clinical *E. coli* isolates in the United States[21,39]. Open circles are the reported data; black line is a logistic model.

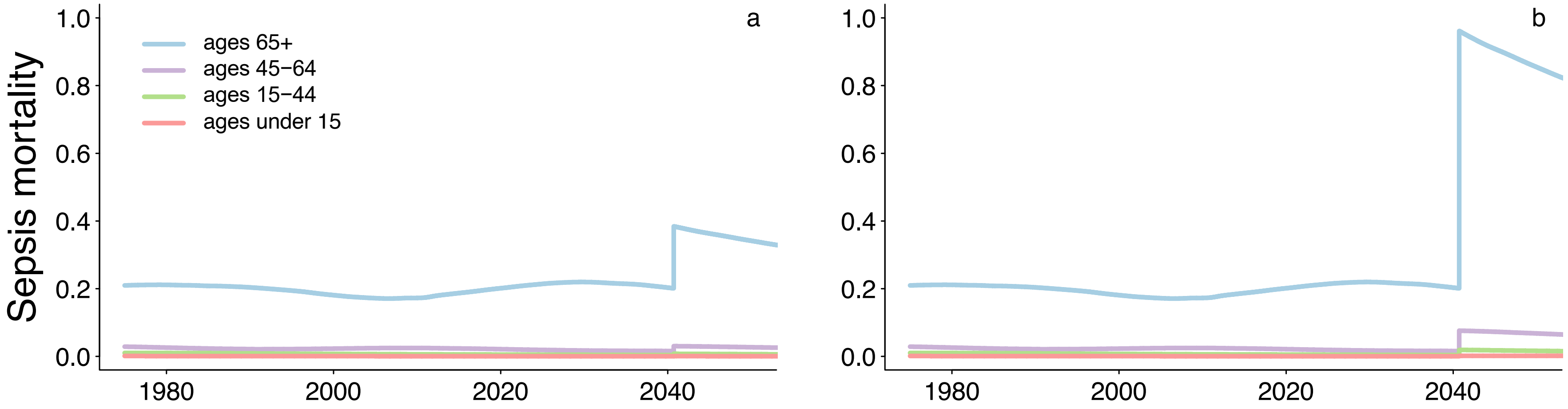

Figure S10. Modeled age class-specific sepsis case mortality rates, assuming the emergence of a pan-resistant bacterial strain. Modeled age class-specific sepsis mortality rates increased sharply with the emergence of a hypothetical pan-resistant *E. coli* strain in 2040. We bracketed the uncertainty of mortality risk associated with a pan-resistant *E. coli* strain by using a low (a) and a high (b) estimate of the increase in mortality due to the absence of any effective antibiotics. These estimated increases in mortality were based on clinical data of temporal delays in broad-spectrum antibiotic administration (see main text for details).
